# Giant cell reparative granuloma in the mandible

**DOI:** 10.1590/S1808-86942010000200022

**Published:** 2015-10-19

**Authors:** Scheila Maria Gambeta Sass, Marlene Corrêa Pinto, Yasser Jebahi, Lilian Bortolon

**Affiliations:** Physician, second year medical resident in the otorhinolaryngology unit of the Holy House of Mercy (Santa Casa de Misericórdia), Curitiba; Physician, otorhinolaryngologist and head & neck surgeon. Head of the otorhinolaryngology unit of the Santa Casa de Misericórdia, Curitiba, PR; Physician, otorhinolaryngologist, assistant physician of the otorhinolaryngology unit of the Santa Casa de Misericórdia, Curitiba. Fellow in Otology; Physician

**Keywords:** giant cell granuloma, giant cell reparative granuloma, mandible, neoplasm

## INTRODUCTION

Giant cell reparative granulomas (GCRG) are non-neoplastic rapidly expanding and locally destructive lesions typical of facial skeleton bones.^1^^,^^2^ Jaffé first described these lesions in 1953, designating benign lesions of the jaw that were histologically different from true giant cell tumors of long bones.^3^

Mandibular GCRG area rare lesions, comprising less than 7% of all benign bone lesions of the jaw.^4^^,^^5^

## CASE REPORT

AP, a female patient aged 29 years, complained of a mass on the right mandibular region starting six months after extraction of a tooth. There was no local pain or weight loss. The physical examination showed a painless hardened mass fixed to deeper layers, measuring about 4 cm diameter.

**Figure 1:**
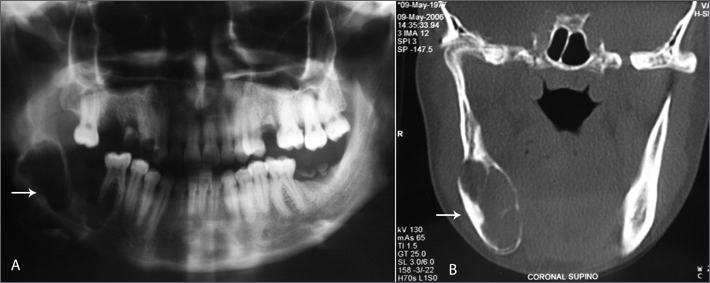
Lytic insuflating lesion with regular borders and fine internal septations, located in the right mandibular body. (A) panoramic mandibular radiograph (B) computed tomography.

A panoramic radiograph of the jaw revealed a well-defined lytic expanding lesion with fine inner septa and no periosteal reaction, located on the right mandibular body (Fig. 1A).

Tomography of the face showed extensive an expanding bone lesion with well-defined borders, bone septa and a calcified matrix, implanted in the alveolar processes (Fig. 1B).

The patient underwent surgical curettage; pathology diagnosed the lesion as a giant cell reparative granuloma. Four months after surgery, a mass was observed on the same site. A second tomography revealed an osteolytic lesion in the right mandible. Surgical curettage was again carried out, and the pathological diagnosis was fibroses, which did not confirm the possibility of recurrence. The patient is being monitored, and no local recurrence has arisen in the first year of follow-up.

## DISCUSSION

GCRG are expanding, rapidly destructive bone lesions located mostly in facial bones; they comprise less than 7% of all benign tumors of the jaw. They may be central when originating directly from bone, or peripheral when its growth originates from soft tissue.^1^^,^^2^

Although the term granuloma is widely used for GCRG, this lesion is neither granulomatous nor reparative from a pathological standpoint; its behavior is neoplastic.^3^

These tumors generally occur in the first to third decades, affecting preferably females (2.1:1).^1^, ^2^, ^3^, ^4^ The clinical features of GCRG of the jaw range from slow asymptomatic growth - as in the case above - to recurring clinically aggressive and painful processes.^4^

The etiology remains controversial. Jaffé's original hypothesis (1953) that the tumor was a hyperplasic reaction to trauma has been abandoned.^3^^,^^5^ In the present case, the patient had a history of dental extraction; generally, however, there are no preceding injuries.

Radiology presents certain specific findings, albeit not pathognomonic. Computed tomography may reveal a uniloculated or multiloculated osteolytic lesion with septa and calcifications in the matrix.^3^^,^^4^

The differential diagnosis is made with the brown tumor of hyperparathyroidism, aneurismatic bone cysts, and mainly with the true giant cell tumor.6 Histologically, true giant cell tumors have little or no intercellular material; giant cells are uniformly distributed, containing many nucleoli and a sparse cytoplasm. GCRG contain less uniformly arranged giant cells with fewer nucleoli and more cytoplasm, which is closely intertwined with microcysts or areas of hemorrhage.^1^ The treatment of choice is surgical curettage of the involved area; the recurrence rate ranges from 10 to 15%. Combined radiotherapy is effective, but is usually applied in difficult cases because there is a risk that radiation-induced osteogenic sarcoma may develop.^3^^,^^4^^,^^5^ In the present case the lesion did not recur; a second curettage was done to clear this doubt. Radiotherapy was excluded because of the risk of malignancy.

## FINAL COMMENTS

GCRG should be considered in the diagnosis of slowly growing increased volume of the jaw in young patients.
